# Immediate *versus* delayed frozen embryo transfer in women following a failed IVF-ET attempt: a multicenter randomized controlled trial

**DOI:** 10.1186/s12958-021-00819-9

**Published:** 2021-08-30

**Authors:** Jing-Yan Song, Feng-Yi Dong, Li Li, Xing-Xing Zhang, Ai-Juan Wang, Yi Zhang, Dan-Dan Gao, Ji-Mei Xiao, Zhen-Gao Sun

**Affiliations:** 1 The First Clinical College, Shandong University of Traditional Chinese Medicine, Jinan, Shandong China; 2 Reproductive and Genetic Center of Integrated Medicine, The Affiliated Hospital of Shandong University of Traditional Chinese Medicine, Jinan, China; 3 Child Rehabilitation Center, Jinan Maternity and Child Care Hospital Affiliated to Shandong First Medical University, Jinan, China; 4 Center for Reproductive Medicine, Maternity and Child Health Care of Zaozhuang, Zaozhuang, China; 5 Reproductive Medical Center, The Second Hospital Affiliated to Shandong University of Traditional Chinese Medicine, Jinan, China; 6 College of Traditional Chinese Medicine, Shandong University of Traditional Chinese Medicine, Jinan, China; 7 Reproductive and Genetic Center, Heze Hospital of Traditional Chinese Medicine, Jinan, China

**Keywords:** Infertility, Frozen embryo transfer, In vitro fertilization, Ongoing pregnancy, Psychological stress

## Abstract

**Background:**

The optimal time at which to perform a frozen-thawed embryo transfer (FET) following a failed *in-vitro* fertilization-embryo transfer (IVF-ET) attempt remains elusive to most reproductive experts. Physicians often delay the introduction of FET due to concerns related to potential residual effects of ovarian hyperstimulation which may interfere with the regular menstrual cycle. Moreover, given that most of the published studies on the topic are retrospective and have inconsistent findings, it is crucial to develop evidence-based randomized control guides for clinical practice. Therefore, this well-designed randomized controlled trial (RCT) was conducted to determine whether it is necessary to delay FET for at least one menstrual cycle after the failure of fresh embryo transfer.

**Methods:**

Infertile women eligible for IVF-ET were invited to participate in this multicenter, randomized, non-inferiority, parallel-group, unblinded, controlled trial at the academic fertility centers of four public hospitals in Chinese Mainland. Infertile women scheduled to receive their first FET cycle after a failed IVF-ET attempt were randomly assigned to either (a) the immediate FET group in which FET was performed in the first menstrual cycle following the failed IVF-ET cycle (n = 366) or (b) the delayed FET group in which FET was performed in the second or subsequent menstrual cycle following the failed IVF-ET cycle (n = 366). All FET cycles were performed during hormone replacement cycles for endometrial preparation. The primary outcome was the ongoing pregnancy, defined as a detectable fetal heart beat beyond twelve weeks of gestation. Secondary outcomes were other pregnancy-related outcomes, maternal and neonatal complications. Analysis was performed by both intention-to-treat and per-protocol principles.

**Results:**

A total of 646 FETs were completed. The frequency of moderate to severe depression and high stress level prior to FET in delayed FET group were significantly higher than that in immediate FET group (10.6% vs 6.1%, *p* = 0.039; 30.3% vs 22.4%, *p* = 0.022, respectively). Immediate FET resulted in a higher frequency of clinical pregnancy than did delayed FET (41.7% vs 34.1%), for a relative risk (RR) of 1.23 (95% confidence interval [CI], 1.00–1.50; *p* = 0.045). Women who underwent immediate FET also had a lower frequency of biochemical pregnancy loss (11.7% vs. 30.6%), with a RR of 0.28 (95% CI 0.23–0.63, *p* < 0.001), and a higher frequency of embryo implantation (25.2% vs. 20.2%), with a RR of 1.25 (95% CI 1.01–1.53; *p* = 0.038). Although the ongoing pregnancy and live birth rates did not differ significantly between the immediate FET and delayed FET groups (37.1% vs 30.3%, RR 1.22, 95% CI 0.99–1.52, *p* = 0.067; 36.5% vs 30.0%, RR 1.22, 95% CI 0.98–1.52, *p* = 0.079, respectively), a multivariate logistic regression analysis adjusted for potential confounders such as depression and stress levels revealed that the immediate FET group had a significantly higher ongoing pregnancy and live birth rates than the delayed FET group (odds ratio 0.68, 95% CI 0.47–0.99, *p* = 0.041; odds ratio 0.67, 95% CI 0.46–0.96, *p* = 0.031). The risks of maternal and neonatal complications were comparable between the two groups.

**Conclusions:**

In women with a previous failed IVF-ET attempt, immediate FET resulted in higher ongoing pregnancy and live birth rates than delayed FET. These findings warrant caution in the indiscriminate application of a delayed FET strategy when apparent risk of high stress level is perceived.

**Trial registration:**

ChiCTR2000033313.

**Supplementary Information:**

The online version contains supplementary material available at 10.1186/s12958-021-00819-9.

## Background

The last three decades have witnessed growing success with frozen embryo transfer (FET). Currently, deferred embryos and cryopreservation are essential aspects of IVF/intracytoplasmic sperm injection (ICSI) treatment [1, 2]. However, the findings of the perinatal outcomes in fresh or frozen embryo transfers are controversial. Notably, FET has been associated with lower rates of antepartum hemorrhage [3], preterm birth, ectopic pregnancy [4–7], and low birth weight [3, 8–10]. Nonetheless, FETs have been linked to higher rates of placental/hypertensive complications [7], large-for-gestational-age infants [7, 11], and inconclusive perinatal mortality rates [3, 11]. Therefore, given the setbacks, most researchers are skeptical of the overall benefits of FET [12–15]. Moreover, physicians face the question of ovarian hyperstimulation and the long-term effects of subsequent FET treatment [16]. As such, FET is often postponed in an attempt to minimize the possible residual effects of ovarian hyperstimulation on endometrial receptivity [17]. However, research on this area remains limited [18, 19]. Deferrals of the FET treatment by physicians, even with the best of intentions, might frustrate couples.

There is limited evidence on the “perfect” timing for FET following a failed-fresh stimulated IVF cycle. However, two options exist, including i) perform FET during the first cycle (an immediate embryo transfer) ii) postpone for at least one menstrual cycle (a delayed embryo transfer). Two studies showed no differences in live birth rates or clinical pregnancies between delayed and immediate FET following a failed fresh IVF [20, 21]. According to Mass K et al., FET should not be postponed following fresh ET failure [17]. However, according to Volodarsky-Perel et al., FET should be postponed for at least one menstrual cycle after a failed-fresh cycle [22]. Women whose attempt at pregnancy fails after a fresh embryo transfer (ET) during a stimulated IVF cycle often opt to proceed with FET immediately to get pregnant as soon as possible. Delaying FET is considered less patient-friendly as it adds to the stress and anxiety accompanying the IVF treatment. Therefore, given the contradictory reports, we conducted this RCT to provide evidence on ongoing pregnancy rate with immediate versus delayed FET following a failed-fresh cycle. We hypothesised that the pregnancy outcomes in immediate FET were non-inferior to that in delayed FET.

## Methods

### Trial design

This study was a multicenter, randomized, controlled, parallel-group clinical trial conducted at academic fertility centers of four public hospitals in the China Mainland. The study enrolled a total of 732 women with a planned transfer of good-quality vitrified-warmed embryos between May 2020 and July 2021. The study was approved by the ethics committees of the participating hospitals. All couples provided voluntary written informed consents before participation. The study protocol was as previously published [23]. This trial was registered with chictr.org.cn (Identifier: ChiCTR2000033313). Final live birth outcomes were available in July 2021. The data were reviewed and approved by an external Data and Safety Monitoring Board.

### Eligibility criteria

Individuals were screened for eligibility. The inclusion criteria were: women aged 21–43 years at the time of IVF/ICSI treatment, participants undergoing IVF/ICSI treatment with a standard controlled ovarian stimulation (COS) protocol, participants had at least one frozen embryo remaining for transfer and had the initial FET cycle after a failed fresh ET. The exclusion criteria were: women with a body mass index (BMI) ≥ 28 kg/m^2^ [24, 25], women with a natural cycle or mild stimulation for IVF/ICSI treatment, severe ovarian hyperstimulation syndrome (OHSS) during COS, history of recurrent pregnancy loss, previously diagnosed with congenital or acquired uterine abnormalities, undergoing blastocyst biopsy for preimplantation genetic testing (PGT) or preimplantation genetic diagnosis (PGD), in vitro maturation (IVM) carried out, use of donor oocytes, or presence of hydrosalpinx, ovarian endometriosis cyst, or endometrial polyps that were not surgically treated. Eligible women signed written consent forms after counseling.

### Randomization and blinding

Women undergoing the initial FET cycle after a failed-fresh ET cycle were randomized into two groups (immediate FET and delayed FET) according to a computer-generated randomization list. Randomization was conducted 14 days after fresh ET (negative blood β-hCG test) for participants with a failed-fresh ET cycle. In addition, randomization was conducted by the project nurse, who was blinded from the entire recruitment and clinical management of the participants. The participants were allocated to four blocks and randomized using random numbers generated using SPSS software (Version 26.0, IBM Corp., Armonk) and were placed in opaque envelopes. Finally, participants were randomized into one of two groups: 1) The immediate FET group, in which FET was performed in the first menstrual cycle (i.e., first vaginal bleeding after withdrawal of luteal phase support) following a failed-fresh ET; 2) The delayed FET group, in which FET was performed in the first or subsequent spontaneous menstrual cycle following a failed-fresh ET. None of the women in the immediate FET group had a spontaneous menstrual cycle before initiation of endometrial preparation. Due to the nature of the intervention, we did not blind the participants and doctors on the assigned groups. However, the trial outcome assessors were blinded to the intervention.

### Trial protocols

Women underwent IVF/ICSI treatment in the fertility centers as clinically indicated. Standard COS protocol with gonadotrophins was performed using a gonadotrophin-releasing hormone (GnRH) antagonist protocol. Furthermore, a fixed GnRH antagonist (GnRH-ant) (0.25 mg, Cetrorelix; Merck Serono, Darmstadt, Germany) was used together with 112.5–225 IU/day of recombinant FSH (600 IU, Puregon, Merck Sharp & Dohme B.V., Haarlem, Netherlands). The gonadotropin doses were determined based on individual patient characteristics [26–28]. The oocyte retrieval was conducted under ultrasound transvaginal guidance, 34–36 h after triggering with recombinant hCG (250 μg, Ovitrelle®, Merck Serono S.p.A., Italy). After that, conventional IVF/ICSI was carried out depending on the partner’s semen quality per the standard protocols at the centers. Normal fertilization was assessed (a second polar body and two pronuclei) after 16–18 h of conventional insemination or ICSI. Notably, an embryo with at least seven cells (grades I and II) on the third day after oocyte retrieval was defined as good quality. In addition, embryos with at least six cells with fragments < 50% were considered frozen. According to the standard protocol, all "good" embryos were vitrified using the cryopreservation method on the third day.

Hormone replacement treatment (HRT) was used for endometrial preparation. Treatment with estradiol valerate (E_2_, Progynova, Schering AG, Berlin, Germany) was commenced on the third day of the menstrual cycle at 4-6 mg daily for 10–12 days. Moreover, vaginal progesterone (90 mg, 8% Crinone, Merck-Serono, Switzerland) was administered at a dose of 90 mg per day, upon the endometrial layer reaching a thickness of 8 mm as revealed by pelvic ultrasound scanning. FET using three-day-old embryos was also scheduled on the fourth day of commencing treatment with vaginal progesterone. Furthermore, one or two embryos with the best morphology were transferred using a soft embryo-transfer catheter under ultrasound guidance [29, 30]. Finally, the serum β-hCG levels were determined fourteen days following FET. The hormone therapy was stopped when the serum β-hCG was negative. Luteal phase support (using estradiol valerate 6 mg daily and vaginal progesterone gel 90 mg daily) was continued in a transvaginal-ultrasound confirmed pregnancy until ten weeks of gestation. The maternal and neonatal outcomes of the initial FET were obtained through a review of medical records.

### Assessment for depression and stress

Standardized and validated questionnaires were administered to the study participants to assess depression and stress before the FET. The participants were asked to complete self-administered questionnaires determining major depression index [31, 32]. The symptoms were rated on a 6-point Likert scale. The scale determines how long the symptoms have been present during the past 14 days. The scale ranges from 0 (no depression) to 50 (extreme depression). It corresponds to the diagnostic criteria for depression in the Diagnostic and Statistical Manual of Mental Disorders (DSM-IV) (including an additional item of low self-esteem) and the International Classification of Diseases and Related Health Problems (ICD-10). Study participants were categorized into four in accordance to the ICD-10 depression categories: mild (two to three core symptoms and two to three additional symptoms); moderate (two core symptoms and four or more additional symptoms); and severe (three core symptoms and five or more additional symptoms). Participants who did not fulfill any of these criteria were categorized as ‘no depression’.

Emotional stress was assessed using the Cohen's Perceived Stress Scale. The participants completed a 10-item self-report questionnaire consisting of a 5-point Likert scale. The final score ranged from 0 (no stress) to 40 (extreme stress). The PSS scale was designed to assess the degree to which respondents found their lives unpredictable, uncontrollable, or overwhelming [33]. The PSS scale was designed for comparisons between -groups and is not a diagnostic tool. The PSS scores were dichotomized into < 19 or ≥ 19, where the latter represents ‘high stress’ based on a previous publication [34].

### Trial outcomes

The primary outcome was an ongoing pregnancy rate, which included natural conception. Ongoing pregnancy was defined as an intrauterine detectable fetal heartbeat after more than twelve weeks of gestation. Secondary outcomes were positive pregnancy rates, pregnancy loss rates, embryo implantation rates, ectopic pregnancy rates, multiple pregnancy rates, live birth rates, pregnancy-related complications, and obstetric complications. Maternal and neonatal outcomes, including preeclampsia, gestational diabetes, gestational hypertension, preterm delivery, low birth weight, infants born small or large for gestational age, and congenital anomalies, were recorded in pregnancies that continued beyond twenty weeks. Supplementary Table S2 provides definitions of all secondary outcomes.

### Statistical analysis

The trial was designed as a non-inferiority study. The PASS software version 11.0 (NCSS, LLC. Kaysville, Utah, USA.) was used to determine the sample sizes for both groups. Notably, the ongoing pregnancy rate per embryo transfer was about 30% based on data from our reproductive center at the time of the trial design. The sample size calculation revealed that 329 women were to be included in each trial group to provide an 80% power to detect a minimal difference of 10% points between the immediate and delayed FET groups for the primary outcome of ongoing pregnancy (40% vs. 30%) at a two-sided α level of 0.05. Finally, the trial planned to include 732 women, with 366 participants in each arm to account for an expected 10% lost to follow-up.

Per protocol (PP) principle was used for the primary statistical analysis. Primary and secondary outcomes were assessed by comparing outcomes after the initial FET cycle. All women in an intention-to-treat (ITT) analysis were accounted for in the group to which they were randomised, irrespective of whether or not they received the treatment. The PP analysis included all women who adhered strictly to the study protocol. The as-treated analysis included women randomized to the immediate FET group who had immediate FET at the first menstrual cycle after a failed fresh ET and women randomized to the delayed FET group who received delayed FET at the second or subsequent menstrual cycle after a failed fresh ET. The ongoing pregnancy rate was determined, and relative risk was used to determine the difference. PP analyses were also performed for all reproductive outcomes. Continuous data were compared using Student’s t-test, and the results were presented as mean (standard deviation) or median (interquartile range). Categorical data were assessed using χ^2^ analysis and Fisher's exact test for expected frequencies less than five. A two-sided P value of less than 0.05 was considered statistically significant. Data analyses were conducted using SPSS version 26.0 and R statistical package version 4.0.0.

Multivariable logistic regression analysis was performed to determine variables independently associated with ongoing pregnancy, live birth, clinical pregnancy, or positive pregnancy and affecting outcomes. Female gender, age (< 35 yrs., ≥ 35 yrs.), anti-müllerian hormone (AMH) (< 1.2 ng/ml, ≥ 1.2 ng/ml), BMI (< 24 kg/m^2^, ≥ 24 kg/m^2^), follicle-stimulating hormone (FSH) (< 10 UI/L, ≥ 10 UI/L), antral follicle count (AFC) (< 10, ≥ 10), endometrial thickness before FET, No. of oocytes retrieved (≤ 9, > 9), No. of transferred frozen-thawed embryos (single, double), method of fertilization (IVF, ICSI), moderate/severe depression prior to FET (yes, no), and high-stress levels prior to FET (yes, no) were included in the analysis.

## Results

### Enrolled patients and baseline characteristics

**Fig. 1:**
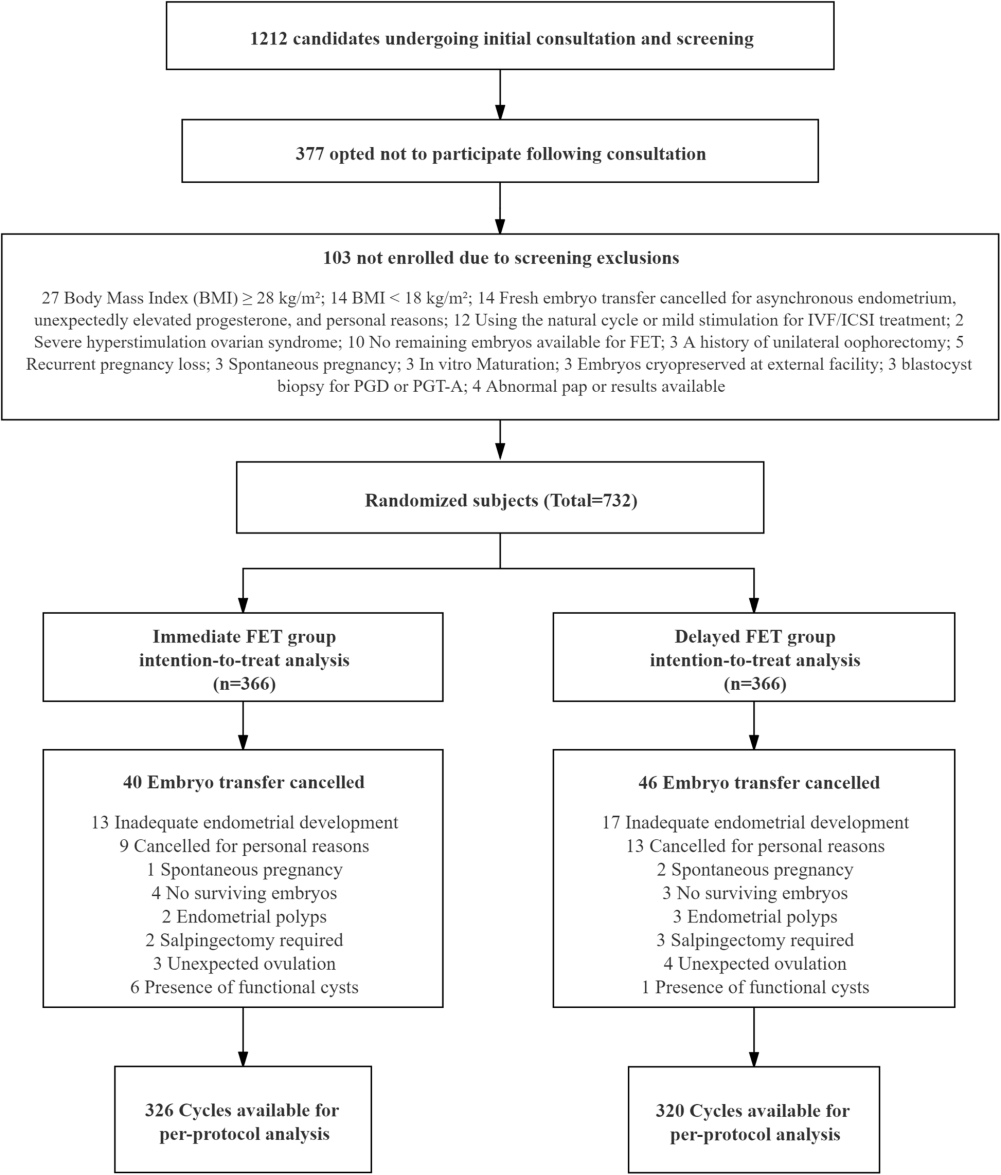
CONSORT diagram: summary of study screening and enrollment, embryo transfer cancellation, and completions per protocol by immediate and delayed FET groups. (FET = frozen-thawed embryo transfer; IVF = in vitro fertilization; ICSI = intracytoplasmic sperm injection; PGD = preimplantation genetic diagnosis; PGT-A = preimplantation genetic testing for aneuploidy)

A total of 1,212 individuals were screened for study enrollment. However, only 732 individuals gave informed consents and were finally enrolled in the study. The participants were randomly assigned in a 1:1 ratio to the immediate or delayed FET groups (**Fig. **1). A total of 732 FET cycles were available for the ITT analysis. Of these, 86 did not undergo embryo transfer or were disqualified per protocol, leaving 646 embryo transfer cycles for the PP analysis. This was commonly due to inadequate endometrial response to estrogen stimulation, n = 30; personal reasons, n = 22; lack of available embryos survival after thawing, n = 7; the presence of endometrial polyps, n = 5; the need for salpingectomy, n = 5; unexpected ovulation, n = 7; the presence of functional cysts, n = 7; or spontaneous pregnancy, n = 3. The canceled embryo transfers or the study disqualifications did not differ significantly between the immediate and delayed FET groups (10.9% vs. 12.6%, *p* = 0.491).

No significant differences in the baseline characteristics (including female gender, age, etiology /duration of infertility, BMI, nulliparity, gravidity, parity, AMH, total AFC, basic FSH, basic luteinizing hormone (LH), and basic estradiol) were observed between the two groups (Table 1). The number of days of COS, total gonadotrophin dose administered, method of fertilization, number of oocytes retrieved, number of embryos available for transfer, number of high-quality day three embryos, and the number of embryos transferred in fresh ET cycle were comparable between the two groups (Table 1). The average number of frozen-thawed embryos transferred (2 vs. 2, *p* = 0.956) and endometrial thickness before FET (10.5 mm vs. 10.3 mm, *p* = 0.388) were comparable between the immediate and delayed FET groups. The average FET time intervals for immediate and delayed FETs were 25.2 days and 103.5 days, respectively. (Table 1).

**Table 1:** Basal demographic and cycle features of FETs which proceeded either within the immediate cycle following OPU (Immediate FET group) or subsequently (Delayed FET group) after a failed-fresh ET. Data are presented as numbers (%) unless otherwise noted

**Characteristic**	**Immediate FET group**	**Delayed FET group**	***P-value***
**Randomized subjects**	**366**	**366**	
Female age at oocyte retrieval (years; mean (SD))	33.8 (3.2)	33.3 (4.1)	*0.062*
Etiology of infertility			*0.815*
Tubal factor	184 (50.3)	177 (48.4)	
Anovulation	43 (11.7)	36 (9.8)	
Male factor	59 (16.1)	63 (17.2)	
Combined factor	68 (18.6)	75 (20.5)	
Unexplained sterility	12 (3.3)	15 (4.1)	
FET interval (days; mean (SD))	25.2 (5.4)	103.5 (23.3)	**< *0.001***
Duration of infertility (years; median (IQR))	4 (4)	5 (2)	*0.817*
Nulliparous	176 (48.1)	180 (49.2)	*0.767*
Gravidity (median (IQR))	1 (2)	1 (2)	*0.978*
Parity (median (IQR))	0 (1)	0 (1)	*0.844*
BMI (kg/m^2^; mean (SD))	23.1 (2.9)	23.5 (2.9)	*0.116*
AMH (ng/ml; median (IQR))	2.9 (1.6)	3.1 (1.2)	*0.354*
Basic FSH (mIU/ml; mean (SD))	6.7 (1.0)	6.8 (1.9)	*0.920*
Basic LH (mIU/ml; mean (SD))	5.0 (1.5)	5.0 (1.2)	*0.688*
Basic estradiol (pg/ml; median (IQR))	35.4 (7.0)	35.3 (8.4)	*0.755*
Total AFC (mean (SD))	10.4 (3.2)	10.7 (2.9)	*0.329*
No of days of COS (mean (SD))	10.7 (2.1)	10.9 (1.8)	*0.132*
Total Gn dose administered (IU; mean (SD))	2140.5 (469.3)	2180.5 (551.2)	*0.291*
Method of fertilization			*0.875*
IVF	247 (67.5)	245 (66.9)	
ICSI	119 (32.5)	121 (33.1)	
No of oocytes retrieved (mean (SD))	10.5 (2.1)	10.7 (2.8)	*0.170*
No of embryos available for transfer (median (IQR))	5.5 (2)	5 (5)	*0.235*
No of high-quality day 3 embryos (median (IQR))	2 (0)	2 (2)	*0.315*
No of embryos transferred in fresh ET cycle (median (IQR))	2 (0)	2 (0)	*0.697*
Single embryo transfer	31 (8.5)	34 (9.3)	
Double embryo transfer	335 (91.5)	332 (90.7)	
No of frozen thawed embryos transferred (median (IQR))	2 (0)	2 (0)	*0.956*
Single embryo transfer	32 (9.8)	31 (9.7)	
Double embryo transfer	294 (90.2)	289 (90.3)	
Endometrial thickness prior to FET (mm; mean (SD))	10.5 (1.8)	10.3 (1.9)	*0.388*
Moderate/severe depression prior to FET (MDI) ^a^	20/326 (6.1)	34/320 (10.6)	***0.039***
High stress level prior to FET (PSS) ^b^	73/326 (22.4)	97/320 (30.3)	***0.022***

*Abbreviation*: *FET* Frozen embryo transfer, *IQR* Interquartile range, *OPU* Ovum pick-up, *Interval* Days elapsed from oocyte retrieval to frozen embryo transfer, *BMI* Body mass index, *AMH* Anti-müllerian hormone, *FSH* Follicle stimulating hormone, *LH* Luteinizing hormone, *AFC* Antral follicle count, *COS* Controlled ovarian stimulation, *IVF* In-vitro fertilization, *ICSI* Intracytoplasmic sperm injection, *Gn* Gonadotropin, *IU* International units, *MDI* Major Depression Index, *PSS* Cohen’s Perceived Stress Scale

^a^ Moderate depression (two core symptoms and four or more additional symptoms); Severe depression (three core symptoms and five or more additional symptoms)

^b^ High stress level defined as ≥ 19 on the PSS scale

### Depression and stress levels before FET

Thirty-four out of 320 women in the delayed FET group (10.6%) had an MDI score corresponding to moderate to severe depression, significantly higher than 6.1% in the immediate FET group (20/326, *p* = 0.039; Table 1). In addition, a total of ninety-seven women out of 320 women in the delayed FET group (30.3%) reported a high-stress level. This was significantly higher than for the immediate FET group, with 22.4% of the women reporting a high-stress level (73 of 326, *p* = 0.022; Table 1).

### Pregnancy and birth outcomes

The primary outcome of ongoing pregnancy rate in the immediate FET group was non-inferior to the delayed FET group after a failed-fresh cycle (33.3% vs. 26.8%, RR 1.25, 95% CI 0.99–1.56, *p* = 0.053 for ITT and 37.1% vs. 30.3%, RR 1.22, 95% CI 0.99–1.52, *p* = 0.067 for PP analysis, respectively). Moreover, no significant difference was found in the live birth rate between the immediate and the delayed FET group (32.8% vs. 26.2%, RR 1.25, 95% CI 0.99–1.57, *p* = 0.052 for ITT analysis and 36.5% vs. 30.0%, RR 1.22, 95% CI 0.98–1.52, *p* = 0.079 for PP analysis, respectively). The results were similar for the ITT and PP analyses (Table 2).

**Table 2:** Pregnancy and birth outcomes compared between the immediate and delayed FET groups. Data are presented as numbers (%)

	**Intention-to-treat analysis**				**Per-protocol analysis**			
	**Immediate FET group**	**Delayed FET group**	**Relative risk (95% CI)**	***P*-value**	**Immediate FET group**	**Delayed FET group**	**Relative risk (95% CI)**	***P*-value**
**Subjects**	**366**	**366**			**326**	**320**		
Positive pregnancy	155 (42.3)	159 (43.4)	0.98 (0.83 to 1.15)	*0.765*	154 (47.2)	157 (49.1)	0.96 (0.82 to 1.13)	*0.643*
Embryo implantation	157/620 (25.3)	125/609 (20.5)	1.23 (1.00 to 1.52)	***0.046***	156/620 (25.2)	123/609 (20.2)	1.25 (1.01 to 1.53)	***0.038***
Clinical pregnancy ^a^	137 (37.4)	111 (30.3)	1.23 (1.01 to 1.51)	***0.042***	136 (41.7)	109 (34.1)	1.23 (1.00 to 1.50)	***0.045***
Biochemical pregnancy loss	18 (11.6)	48 (30.2)	0.39 (0.24 to 0.63)	** < *0.001***	18 (11.7)	48 (30.6)	0.28 (0.23 to 0.63)	** < *0.001***
Clinical pregnancy loss ^b^	15 (10.9)	13 (11.7)	0.94 (0.47 to 1.88)	*0.850*	15 (11.0)	12 (11.0)	1.00 (0.49 to 2.05)	*0.996*
Total pregnancy loss	33 (21.3)	61 (38.4)	0.56 (0.39 to 0.80)	***0.001***	33 (21.4)	60 (38.2)	0.56 (0.39 to 0.81)	***0.001***
Ectopic pregnancy	2 (1.3)	5 (3.1)	0.41 (0.08 to 2.08)	*0.448*	2 (1.3)	5 (3.2)	0.40 (0.08 to 2.04)	*0.448*
Multiple pregnancy	18 (13.1)	14 (12.6)	1.04 (0.54 to 2.00)	*0.902*	18 (13.2)	14 (12.8)	1.03 (0.54 to 1.98)	*0.928*
Ongoing pregnancy	122 (33.3)	98 (26.8)	1.25 (0.99 to 1.56)	*0.053*	121 (37.1)	97 (30.3)	1.22 (0.99 to 1.52)	*0.067*
Live birth	120 (32.8)	96 (26.2)	1.25 (0.99 to 1.57)	*0.052*	119 (36.5)	96 (30.0)	1.22 (0.98 to 1.52)	*0.079*

*Abbreviation*: *FET* Frozen thawed embryo transfer, *CI* Confidence interval

^a^ In the Immediate FET group, one woman conceived naturally and delivered a healthy male infant vaginally

^b^ In the Delayed FET group, two women conceived spontaneously, however, one miscarried in the second trimester because of fetal anomalies

In the immediate FET group, 136 of 326 women (41.7%) had a higher clinical pregnancy rate compared with 109 out of 320 (34.1%) in the delayed FET group (RR 1.23, 95% CI 1.00–1.50, *p* = 0.045 for PP analysis). Furthermore, the ITT and PP analyses yielded the same findings (37.4% vs. 30.3%, RR 1.23, 95% CI 1.01–1.51, *p* = 0.042; Table 2). The embryo implantation rate was 25.2% (156/620) in the immediate FET group and 20.2% (123/609) in the delayed FET group (RR 1.25, 95% CI 1.01–1.53, *p* = 0.038 for PP analysis). Additionally, the results of the ITT analysis were similar to the PP analysis (25.3% vs. 20.5%, RR 1.23, 95% CI 1.00–1.52, *p* = 0.046; Table 2).

Biochemical pregnancy loss was more than two times frequent among individuals in the immediate FET than the delayed FET (30.2% vs.11.6%, *p* < 0.001 for ITT, and 30.6% vs.11.7%, *p* < 0.001 for PP analysis; Table 2). Nonetheless, there was no statistically significant difference in the rate of clinical pregnancy loss between the two groups. Nevertheless, total pregnancy loss per positive hCG test was approximately 80% higher in delayed FET cycles than immediate FET in the ITT analysis (38.4% vs. 21.3%, *p* = 0.001) and the PP analysis (38.2% vs. 21.4%, *p* = 0.001) (Table 2). The hCG positive pregnancy rate, multiple pregnancy rate, and ectopic pregnancy rate did not differ significantly between the two groups (Table 2). The detailed results of secondary outcomes on maternal and perinatal complications are presented in Supplementary Table S1.

A multivariable logistic regression analysis was performed to adjust for potential confounding factors, and the results are presented in Table 3. The timing of initial FET after a failed fresh ET attempt had no significant effect on the positive pregnancy rate. Interestingly, after adjusting for a variety of confounding variables that may influence the success of frozen-thawed embryo transfer, we found that the timing of FET was an independent and significant factor in clinical pregnancy rate, ongoing pregnancy rate, and live birth rate (odds ratio [OR] 0.68, 95% CI 0.47–0.91, *p* = 0.031; OR 0.68, 95% CI 0.47–0.99, *p* = 0.041; OR 0.67, 95% CI 0.46–0.96, *p* = 0.031, respectively; Table 3). The other variables that had a significant effect on ongoing pregnancy rates were BMI, AFC, method of fertilization, and high-stress levels before FET (Table 4).

**Table 3:** Relationship between the timing of initial FET after a fresh IVF-ET attempt failure and pregnancy outcomes in different models

Pregnancy outcomes	Timing of initial FET	Crude model ^a^		Adjusted model I ^b^		Adjusted model II ^c^	
		**OR (95% CI)**	***P* value**	**OR (95% CI)**	***P* value**	**OR (95% CI)**	***P* value**
**Positive pregnancy**	Immediate FET group	Reference		Reference		Reference	
	Delayed FET group	1.09 (0.80to 1.48)	0.587	1.04 (0.75 to 1.44)	0.810	0.95 (0.67 to 1.35)	0.781
**Clinical pregnancy**	Immediate FET group	Reference		Reference		Reference	
	Delayed FET group	0.74 (0.54 to 1.02)	0.066	0.70 (0.50 to 0.97)	**0.031**	0.68 (0.47 to 0.97)	**0.031**
**Ongoing pregnancy**	Immediate FET group	Reference		Reference		Reference	
	Delayed FET group	0.78 (0.55 to 1.05)	0.096	0.71 (0.51 to 0.99)	**0.048**	0.68 (0.47 to 0.99)	**0.041**
**Live birth**	Immediate FET group	Reference		Reference		Reference	
	Delayed FET group	0.75 (0.54 to 1.04)	0.080	0.70 (0.50 to 0.98)	**0.038**	0.67 (0.46 to 0.96)	**0.031**

*Abbreviation*: *AMH* Anti-müllerian hormone, *BMI* Body mass index, *FSH* Follicle stimulating hormone, *OR* Odds ratio, *CI* Confidence interval, *FET* Frozen embryo transfer, *IVF-ET* In vitro fertilization-embryo transfer, *AFC* Antral follicle count, *COS* Controlled ovarian stimulation; Gn, gonadotropin

^a^ No adjustments for other covariates

^b^ Adjusted for female age (< 35 yrs., ≥ 35 yrs.), moderate/severe depression prior to FET (yes, no), and high stress level prior to FET (yes, no)

^c^ Adjusted for female age (< 35 yrs., ≥ 35 yrs.), AMH (< 1.2 ng/ml, ≥ 1.2 ng/ml), BMI (< 24 kg/m^2^, ≥ 24 kg/m^2^), basic FSH (< 10 UI/L, ≥ 10 UI/L), AFC (< 10, ≥ 10), endometrial thickness before FET, No of oocytes retrieved (≤ 9, > 9), No of transferred frozen thawed embryos (single, double), method of fertilization (IVF, ICSI), moderate/severe depression prior to FET (yes, no), and high stress level prior to FET (yes, no)

**Table 4:** Crude and adjusted odds ratio (OR) for timing of initial frozen embryo transfer (FET) after a failed fresh IVF-ET attempt and other potential confounders for ongoing pregnancy

Variable	Crude OR (95% CI)	Adjusted OR (95% CI)
Timing of initial FET		
Immediate	Reference	Reference
Delayed	0.78 (0.55 to 1.05)	0.68 (0.47 to 0.99)
Female age		
< 35 yrs	Reference	Reference
≥ 35 yrs	0.77 (0.52 to 1.12)	0.87 (0.58 to 1.31)
Anti-müllerian hormone (AMH)		
< 1.2 ng/ml	Reference	Reference
≥ 1.2 ng/ml	1.12 (0.68 to 1.83)	1.20 (0.71 to 2.03)
Body mass index (BMI)		
< 24 kg/m^2^	Reference	Reference
≥ 24 kg/m^2^	0.67 (0.48 to 0.94)	0.62 (0.43 to 0.89)
Basic FSH		
< 10 UI/L	Reference	Reference
≥ 10 UI/L	0.84 (0.56 to 1.26)	0.90 (0.58 to 1.40)
Antral follicle count (AFC)		
< 10	Reference	Reference
≥ 10	1.31 (0.94 to 1.83)	1.49 (1.05 to 2.12)
No of oocytes retrieved		
≤ 9	Reference	Reference
> 9	1.25 (0.86 to 1.82)	1.33 (0.88 to 2.02)
Method of fertilization		
IVF	Reference	Reference
ICSI	0.61 (0.43 to 0.87)	0.65 (0.45 to 0.95)
No of transferred frozen thawed embryos		
Single	Reference	Reference
Double	1.68 (0.92 to 3.08)	1.33 (0.70 to 2.53)
Endometrial thickness before FET	1.09 (0.99 to 1.19)	1.05 (0.95 to 1.16)
Moderate/severe depression prior to FET		
No	Reference	Reference
Yes	0.75 (0.40 to 1.39)	0.70 (0.36 to 1.36)
High stress level prior to FET		
No	Reference	Reference
Yes	0.27 (0.17 to 0.43)	0.28 (0.17 to 0.45)

*Abbreviation*: *IVF-ET* In vitro fertilization-embryo transfer, *CI* Confidence interval

### Safety outcomes

Overall, there were no apparent safety concerns. Eleven serious adverse events were reported among the study population, all of which were judged to be unrelated or unlikely to be related to the investigational procedure. These adverse events were hyperemesis (n = 1), ovarian torsion (n = 1), acute appendicitis (n = 1), heterotopic pregnancy (n = 7), and fetal malformations (n = 1).

## Discussion

The results of this RCT revealed significantly higher embryo implantation and clinical pregnancy rates and a lower biochemical pregnancy loss rate in the immediate FET group than the delayed FET group. Although no significant differences in ongoing pregnancy and live birth rates were detected between the two groups, the binary multivariate logistic regression analysis revealed a significant difference in favor of the immediate FET group. The high-stress level before FET was also shown to affect the ongoing pregnancy rate.

FET has been increasingly performed in assisted reproductive technologies (ART) in the last few decades [1]. The development of controlled ovarian stimulation (COS) protocol has led to increased embryo freezing techniques and surplus embryos. The freeze-all policy was also established to overcome potential adverse carryover effects of COS [2, 14]. However, two recent large randomized controlled studies did not find any significant difference between fresh ET and FET among infertile ovulatory women [35] or women without polycystic ovaries [36]. Therefore, some scholars argue that the residual effect of COS on endometrial receptivity on the next menstrual cycle is nonexistent. In addition, delayed FET could emotionally stress patients who may be eager to conceive as soon as possible, especially after a failed fresh ET cycle and, may lead to drop-out from infertility treatment [37]. Therefore, there is a need to avail evidence on the perfect timing of FET after COS.

Four previously conducted studies showed no difference in the live birth rate between delayed FET and immediate FET groups following a failed-fresh cycle [17, 20, 21, 37]. However, a study conducted by Volodarsky-Perel et al., using only GnRH agonist long protocol, reported contrasting results. The hormonal profile and function of the corpus luteum after oocyte pick-up differed between the GnRH agonist long protocol and GnRH antagonist protocol [22]. GnRH receptors were downregulated in the GnRH agonist long protocol, and the recovery of GnRH receptors took longer than the GnRH antagonist protocol. Also, hCG used in GnRH agonist long protocol had a longer half-life and could have impacted the function of the corpus luteal and endometrial receptivity of the subsequent cycle. Based on published literature, immediate FET after a failed fresh cycle was assumed to have less harm than delayed FET except in GnRH long protocol. However, these studies had several limitations. A study by Huang et al. did not include an adequate number of patients [37]. In addition, a study by Horowitz et al. had a possibility of practical bias [21].

A recent RCT showed that immediate FET improved ongoing pregnancy rates (47.2% vs. 39.3%, *p* = 0.03) and reduced the risk of miscarriage (11.2 vs. 19.7%, *p* = 0.02) [38]. However, this study was limited as it included patients with a previous failed fresh ET and those who performed a freeze-all strategy. Moreover, the study had an unequal balance of subjects in terms of female age and number of oocytes retrieved, despite randomization. Furthermore, the RCT did not assess for depression and stress levels, thus it is unknown whether women who undergo delayed FET are more stressed than women who undergo immediate FET. A recent study determining attitudes towards elective FET in a freeze-all strategy showed that 59.2% of women and 59.7% of men would choose elective FET over fresh ET if the chances of pregnancy were the same [39]. Undergoing a delayed FET cycle is an emotional process for couples who have already experienced a delay in conception, especially for women who have experienced a failed fresh ET cycle. Delaying FET may be a potential source of ART-related stress to patients and a reason for treatment discontinuation [40]. Thus, one possible explanation for our results is the negative emotional state such as stress or depression of the delayed FET group.

In the present study, the frequency of moderate to severe depression and high-stress levels before FET in the delayed FET group was significantly higher than that in the immediate FET group. Moreover, logistic regression analysis also revealed the adverse effects of high-stress levels on ongoing pregnancy and live birth rates. Therefore, immediate FET is beneficial to alleviate psychological pressure on patients and shorten the time of pregnancy. When female mouse embryos were exposed with stress, the frequency of implantations dropped substantially and the percentage of apoptotic cells in blastocysts rose [41, 42]. This may be because the stress response altered the endometrium's oxidative stress pathway, impairing endometrial remodeling; the increased production of oxidative stress molecules in serum and oviduct also impairs embryo development [43]. Other mechanisms may include a more favorable endometrial immune status after COS [44], while the corpora lutea producing high levels of vasodilatory and angiogenic factors during stimulation [45] could also have a positive effect in case of an immediate FET. However, it is not obvious that the multiple corpus luteum could produce a higher serum level of relaxin [46]. Further, it is not clear if higher serum levels of relaxin in a COS cycle could continue to the subsequent menstrual cycle. Therefore, further prospective clinical and translational studies are necessary to validate these findings and investigate the underlying mechanisms.

To the best of our knowledge, this is the first RCT determining the timing of FET following a failed fresh IVF-ET cycle. However, this study had some limitations. The primary outcome was the ongoing pregnancy rate determined as an intrauterine detectable fetal heartbeat after more than twelve gestational weeks. However, the live birth rate was also reported. We caution that this study only evaluated the effect of delayed and immediate FET on the ongoing pregnancy rate following HRT; thus, the results may not be extrapolated to other methods of endometrial preparation, such as natural cycle FET. Additionally, the multiple pregnancy rate remained high at more than 10% after the transfer of a maximum of two embryos. In the future, randomized studies comparing the live birth rate as the primary outcome following a single blastocyst transfer are needed.

## Conclusions

In conclusion, the immediate FET group resulted in higher ongoing pregnancy and live birth rates and a lower biochemical pregnancy loss rate than the delayed FET group. These findings warrant caution in indiscriminately using delayed FET in patients with high-risk stress levels. This study recommends immediate FET following a failed fresh IVF-ET cycle to improve the live birth rate.

## Supplementary Information

**Additional file 1: Table S1**. Maternal and perinatal complications of the initial FET after a failed fresh IVF-ET attempt. Values are number/total number (%) unless stated otherwise. **Table S2**. The definitions of secondary outcomes.

## Data Availability

The data generated and analyzed in this study will be availed upon request by the corresponding author.
