# Supplementary material for: Immediate versus delayed frozen embryo transfer in women following a failed IVF-ET attempt: a multicenter randomized controlled trial
Source: Reprod Biol Endocrinol. 2021 Aug 30;19:131. doi: 10.1186/s12958-021-00819-9 (PMC8404351; doi:10.1186/s12958-021-00819-9)
Supplement: Supplementary file 1 — Additional file 1: Table S1. Maternal and perinatal complications of the initial FET after a failed fresh IVF-ET attempt. Values are number/total number (%) unless stated otherwise. Table S2. The definitions of secondary outcomes. [file 12958_2021_819_MOESM1_ESM.docx]

| **Table S1** Maternal and perinatal complications of the initial FET after a failed fresh IVF-ET attempt. Values are number/total number (%) unless stated otherwise | | | | | | |
| --- | --- | --- | --- | --- | --- | --- |
|  | **Intention-to-treat analysis** | | | **Per-protocol analysis** | | |
|  | **Immediate FET group** | **Delayed FET group** | **P-value** | **Immediate FET group** | **Delayed FET group** | **P-value** |
| **Subjects** | **366** | **366** |  | **326** | **320** |  |
| Preeclampsia among clinical pregnancies | 10/137 (7.3) | 13/111 (11.7) | *0.234* | 10/136 (7.4) | 12/109 (11.0) | *0.223* |
| Gestational diabetes among clinical pregnancies | 3/137 (2.2) | 2/111 (1.8) | *1.000* | 3/136 (2.2) | 2/109 (1.8) | *1.000* |
| Gestational hypertension among clinical pregnancies | 7/137 (5.1) | 7/111 (6.3) | *0.685* | 7/136 (5.1) | 7/109 (6.4) | *0.669* |
| Preterm delivery among clinical pregnancies | 5/137 (3.6) | 8/111 (7.3) | *0.211* | 5/136 (3.7) | 7/109 (6.4) | *0.322* |
| Low birth weight ¶ | 8/120 (6.7) | 10/96 (10.4) | *0.322* | 8/119 (6.7) | 9/96 (9.4) | *0.474* |
| Small for gestational age § | 9/120 (7.5) | 5/96 (5.2) | *0.497* | 9/119 (7.6) | 5/96 (5.2) | *0.487* |
| Large for gestational age § | 9/120 (7.5) | 3/96 (3.1) | *0.163* | 9/119 (7.6) | 3/96 (3.1) | *0.159* |
| Congenital anomalies ‡ | 2/120 (1.7) | 2/96 (2.1) | *1.000* | 2/119 (1.7) | 1/96 (1.0) | *1.000* |
| Abbreviation: IVF-ET, in vitro fertilization-embryo transfer; FET, frozen thawed embryo transfer.  ¶ Low birth weight was defined as birth weight of less than 2500 g.  § Small and large for gestational age defined as less than or more than two standard deviation units from expected birth weight. Small and large for gestational age were calculated from growth curves for Scandinavian children adjusted for sex and gestational age.  ‡ The denominator for congenital anomalies includes the number of live newborns plus the number of fetuses that were therapeutically terminated. | | | | | | |

| **Table S2** The definitions of secondary outcomes. | |
| --- | --- |
| **Outcomes** | **Definitions** |
| Positive pregnancy | Positive pregnancy (biochemical pregnancy), i.e. serum β-hCG level ≥ 10mIU/mL, 14 days after embryo transfer. |
| Embryo implantation rate | Embryo implantation rate was defined as the number of intrauterine gestational sacs observed divided by the number of embryos transferred. |
| Clinical pregnancy | Clinical pregnancy, defined as an intrauterine gestational sac with fetal heartbeat detected by transvaginal ultrasonography after 6 weeks of gestation. |
| Ectopic pregnancy | Ectopic pregnancy, defined as a pregnancy in which implantation takes place outside the uterine cavity. |
| Clinical pregnancy loss | Pregnancy loss, defined as clinically recognized spontaneous loss of pregnancy before the completion of twenty gestational weeks. |
| Biochemical pregnancy loss | Non-visualized pregnancy losses documented only by a positive pregnancy test (serum or urine human chorionic gonadotrophin). |
| multiple pregnancies | A pregnancy in which two or more embryos develop in the uterus at the same time. |
| Live birth | Live birth, defined as the birth of at least one child with breath and heartbeat, irrespective of the duration of gestation. |
| Preeclampsia | It is characterized by hypertension (blood pressure [BP] > 140/90) and proteinuria (300 mg in 24 hours or 1+ dipstick) after 20 weeks’ gestation. |
| Gestational diabetes | Diagnosis is made when fasting plasma glucose is measured at 24-28 weeks of gestation and an oral glucose tolerance test (OGTT) is performed, and normal criteria are met or exceeded. |
| Gestational hypertension | Gestational hypertension: blood pressure ≥ 140/90 mmHg after 20 weeks of gestation with negative urine protein or 24-hour urine protein quantification < 0.3 g/L. |
| Preterm delivery | Preterm delivery refers to delivery between 28 weeks and less than 37 weeks of gestation. |
| Low birth weight | Low birth weight, defined as < 2,500 g of a newborn’s weight. |
| Small for gestational age (SGA) | The most common definition of small for gestational age (SGA) is a birth weight that is below the 10th percentile. |
| Large for gestational age (LGA) | LGA refers to neonatal birth weight larger than the 90th percentile for a given gestational age. |
| Congenital anomalies | A congenital anomaly is a medically diagnosed condition present at or from birth that significantly deviates from the common structure or function of the body. |
